# Supplementary material for: The Reentry Helix Is Potentially Involved in Cholesterol Sensing of the ABCG1 Transporter Protein
Source: Int J Mol Sci. 2022 Nov 8;23(22):13744. doi: 10.3390/ijms232213744 (PMC9698493; doi:10.3390/ijms232213744)
Supplement: Supplementary file 1 [file ijms-23-13744-s001.zip › ijms-1957456-supplementary.pdf]

International Journal of *Molecular Sciences*

MDPI

Supplementary Material

The reentry helix is potentially involved in cholesterol sensing of the ABCG1 transporter protein

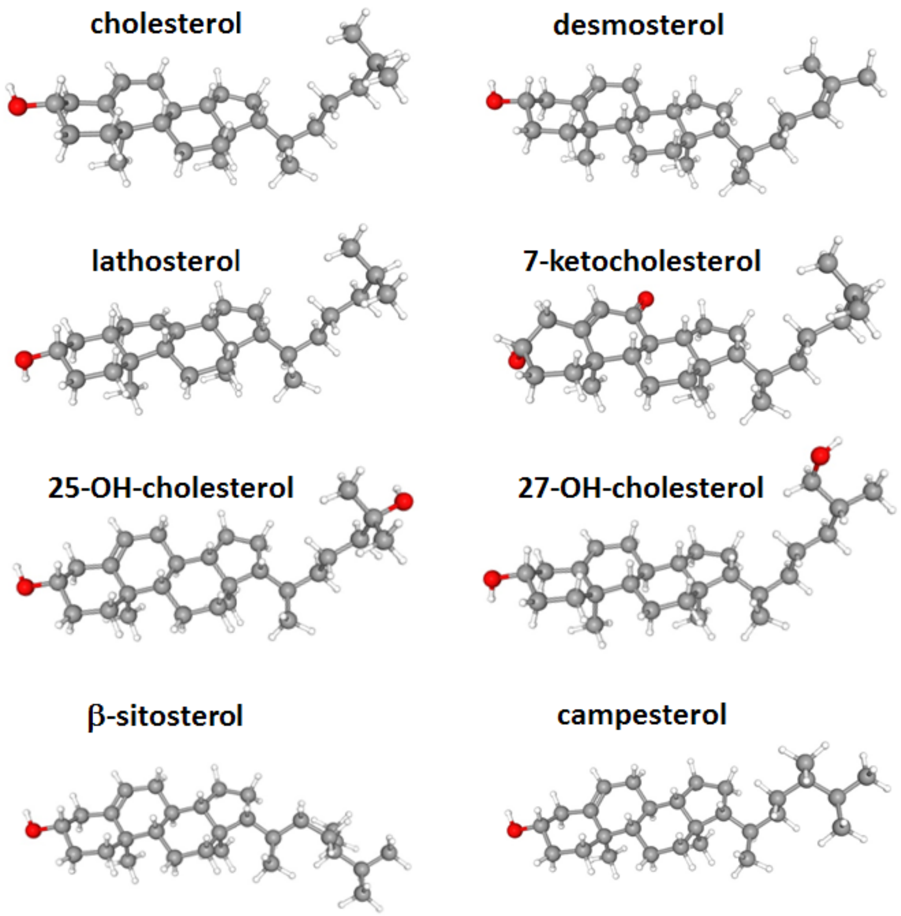

Supplementary Figure S1. Chemical strictures of the studied sterol compounds. Sticks and balls representations of various sterols are form PubChem (https://pubchem.ncbi.nlm.nih.gov/). CIDs are as follows: cholesterol – 5997; desmosterol – 439577; lathosterol – 65728; 7-ketocholesterol – 91474; 25-hydroxycholesterol – 65094; 27-hydroxycholesterol – 123976; β-sitosterol – 222284; campesterol – 173183. Carbon and hydrogen atoms are represented by gray and white balls, respectively, whereas oxygen atoms are indicated by red balls.

1 MACLMAAFSV GTAMNASSYS AEMTEPKSVC VSVDEVVSSN MEATETDLLN GHLKKVDNNL
61 TEAQRFSSLP RRAAVNIEFR DLSYSVPEGP WWRKKGYKTL LKGISGKFNS GELVAIMGPS
121 GAGKSTLMNI LAGYRETGMK GAVLINGLPR DLRCFRKVSC YIMQDDMLLP HLTVQEAMMV
181 SAHLKLQEKD EGRREMVKEI LTALGLLSCA NTRTGSLSGG QRKRLAIALE LVNNPPVMFF
241 DEPTSGLDSA SCFQVVSLMK GLAQGGRSII CTIHQPSAKL FELFDQLYVL SQGQCVYRGK
301 VCNLVPYLRD LGLNCPTYHN PADFVMEVAS GEYGDQNSRL VRAVREGMCD SDHKRDLGGD
361 AEVNPFLWHR PSEEDSSSME GCHSFSASCL TQFCILFKRT FLSIMRDSVL THLRITSHIG
421 IGL**LIGLLYL** GIGNEAKKVL SNSGFLFFSM LFLMFAALMP TVLTFPLEMG VFLREHLNYW
481 YSLKAYYLAK TMADVPFQIM FPVAYCSIVY WMTSQPSDAV RFVLFAALGT MTSLVAQSLG
541 LLIGAASTSL QVATFVGPVT AIPVLLF**SGF FVSF**DTIPTY **LQWMSYISYV R**YGFEGVILS
601 IYGLDREDLH CDIDETCHFQ KSEAI**LREL**D VENAKLYLDF IVLGIFFIS**L RLIAYFVLRY**
661 **KIRAER**

Supplementary Figure S2. Cholesterol-interacting sequences in ABCG1. Amino acid sequence of the short isoform of human ABCG1 with the studied Cholesterol Recognition Amino acid Consensus (CRAC) and Steroid-Binding Element (SBE) motifs highlighted. The consensus sequences for CRAC and SBE motifs are L/V-X_{1-5}-Y-X_{1-5}-R/K and (L/M-XX-)L-XX-L, respectively.

ABCG1 variant							mean	SEM
**wt**	basal	19.79	26.19	15.32	20.36		**20.41**	**2.23**
	Chol	39.01	45.40	49.74	45.63		**44.94**	**2.22**
	Rhod123	39.99	37.72	43.19	41.29		**40.55**	**1.15**
**L430A**	basal	17.08	16.51	16.55	20.89		**17.76**	**1.05**
	Chol	26.62	25.53	24.12	34.20		**27.62**	**2.25**
	Rhod123	24.62	25.53	25.53	31.82		**26.88**	**1.66**
**F571A**	basal	17.24	23.74	13.08			**18.02**	**3.10**
	Chol	19.53	22.46	14.59			**18.86**	**2.30**
	Rhod123	16.30	18.17	7.96			**14.14**	**3.14**
**L626A**	basal	30.28	19.60	14.07			**21.32**	**4.76**
	Chol	29.21	17.03	14.02			**20.09**	**4.64**
	Rhod123	29.88	18.74	12.05			**20.22**	**5.20**
**Y586A**	basal	9.47	7.63	7.15	4.77		**7.25**	**0.97**
	Chol	15.69	12.07	9.96	6.73		**11.11**	**1.88**
	Rhod123	12.86	8.05	7.29	9.11		**9.33**	**1.23**
**Y655A**	basal	16.81	8.15	10.01	4.47		**9.86**	**2.59**
	Chol	18.28	9.01	10.57	5.19		**10.76**	**2.75**
	Rhod123	18.55	10.15	11.56	4.90		**11.29**	**2.81**
**Y660A**	basal	23.69	27.96		20.47		**24.04**	**2.17**
	Chol	44.61	47.28		35.64		**42.51**	**3.52**
	Rhod123	43.91	51.94		42.48		**46.11**	**2.94**
**β-gal**	basal	10.19	6.29	2.43	3.57	7.74	**6.04**	**1.40**
	Chol			4.43	4.14	10.81	**6.46**	**2.18**
	Rhod123			2.28	3.28	11.68	**5.75**	**2.98**

Supplementary Table S1. Parallel measurements of ATPase activity of various ABCG1 variants. The vanadate-sensitive ATPase activities of Sf9 cell membrane preparations containing the indicated ABCG1 variants were determined in the absence (basal) and presence of cyclodextrin-complexed cholesterol (Chol), as well as in the presence of rhodamine 123 (Rhod123). For negative control, Sf9 cell membrane preparations containing β-galactosidase (β-gal) were used. The values represent the vanadate-sensitive ATPase activities in nmol Pi/mg protein/min units.

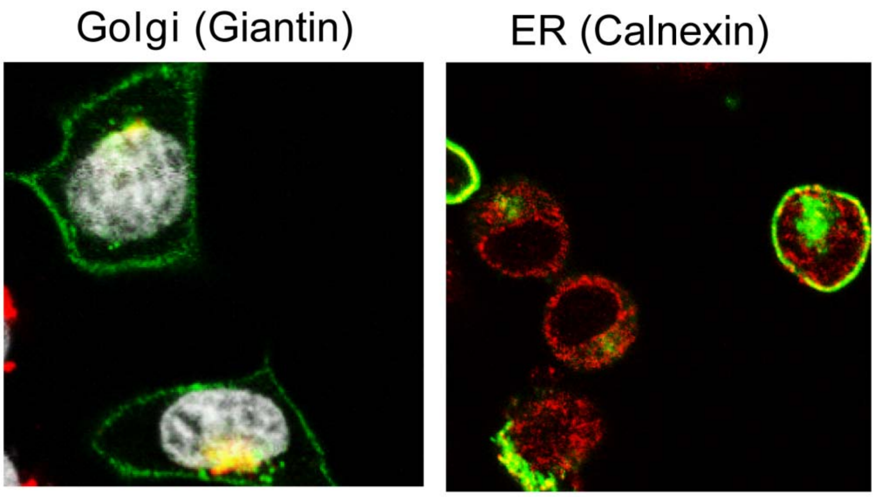

Supplementary Figure S3. Subcellular localization of ABCG1. HeLa cells were transfected with the short isoform of ABCG1 (G1-S) and immunostained with antibodies against ABCG1 (green) and cellular markers, such as giantin or calnexin (red) for the Golgi apparatus or endoplasmic reticulum, respectively. On the left panel the cell nuclei were also counterstained with Hoechst 33342 dye (grey).

HeLa	12/10	01/14	01/21	01/28	02/25	03/04	05/13	05/14	05/15	07/29	mean	SEM
**WT**	19.29	10.05	9.79	10.39	12.69	29.74	26.00	23.93	29.76	27.17	**19.88**	**2.67**
**G1KM**	3.40	6.40	5.72	3.26	3.39	4.55	4.23	9.42	9.62	10.83	**6.08**	**0.91**
**L430A**	20.95	22.71	11.70	12.03	9.73	19.95	21.08	27.23	36.50	39.31	**22.12**	**3.16**
**F571A**	6.89	7.86	6.39	4.80	5.24	3.47	6.22	12.78	16.58	15.32	**8.55**	**1.46**
**L626A**	4.63	7.57	5.98	3.56	7.21	3.63	5.00	8.57	9.57	17.01	**7.27**	**1.26**
**Y586A**	4.97	4.35	2.42	2.02	5.78	2.60	2.12	11.09	5.30	8.99	**4.96**	**0.96**
**Y655A**	3.60	7.19	5.94	6.62	3.24	4.14	2.30	11.48	5.87	11.17	**6.16**	**0.99**
**Y660A**	14.15	12.16	9.10	2.72	5.57	8.75	2.38	22.70	20.37	25.49	**12.34**	**2.60**
**G1-L**	n.d.	13.93	6.69	10.74	9.83	18.36	37.79	21.21	29.08	28.05	**19.52**	**3.47**
**G1-LKM**	n.d.	6.59	3.87	1.98	5.58	3.67	2.11	7.53	4.68	10.03	**5.12**	**0.87**
**HEK293**		**01/14**	**01/21**	**01/28**	**02/25**	**03/04**	**05/13**	**05/14**	**05/15**		**mean**	**SEM**
**WT**	n.d.	17.10	8.34	5.15	12.20	8.17	4.19	3.27	1.41	n.d.	**7.48**	**1.83**
**G1KM**	n.d.	5.51	3.06	2.34	2.44	1.81	0.97	1.45	0.33	n.d.	**2.24**	**0.56**
**L430A**	n.d.	7.56	3.75	7.67	12.93	13.53	2.75	9.25	4.64	n.d.	**7.76**	**1.42**
**F571A**	n.d.	4.09	2.91	13.16	7.68	5.65	1.50	7.02	3.10	n.d.	**5.64**	**1.31**
**L626A**	n.d.	3.93	2.61	5.48	0.40	3.86	1.20	4.27	0.87	n.d.	**2.82**	**0.65**
**Y586A**	n.d.	4.77	2.74	4.60	8.51	4.22	3.18	6.30	1.54	n.d.	**4.48**	**0.77**
**Y655A**	n.d.	6.10	2.59	1.16	3.10	2.78	1.14	2.90	0.41	n.d.	**2.52**	**0.62**
**Y660A**	n.d.	5.77	3.27	10.61	7.66	5.78	1.24	4.87	2.65	n.d.	**5.23**	**1.05**
**G1-L**	n.d.	8.26	7.01	7.43	10.54	7.33	1.90	7.43	5.04	n.d.	**6.87**	**0.89**
**G1-LKM**	n.d.	2.45	5.28	2.21	1.52	2.13	0.85	4.47	0.53	n.d.	**2.43**	**0.59**

Supplementary Table S2. Parallel measurements of apoptotic effect of various ABCG1 mutants. Values represent the fraction of the apoptotic cells (%) in HeLa and HEK293 cell cultures transiently transfected with the indicated ABCG1 variant. Apoptotic cells were identified by Annexin V binding. For positive and negative controls, the wild type of the short isoform (WT) and its catalytically inactive variant (G1KM) were used respectively. For comparison the apoptotic effects of the long isoform (G1-L) and its inactive mutant variant (G1-LKM) were also assessed. n.d. – not determined

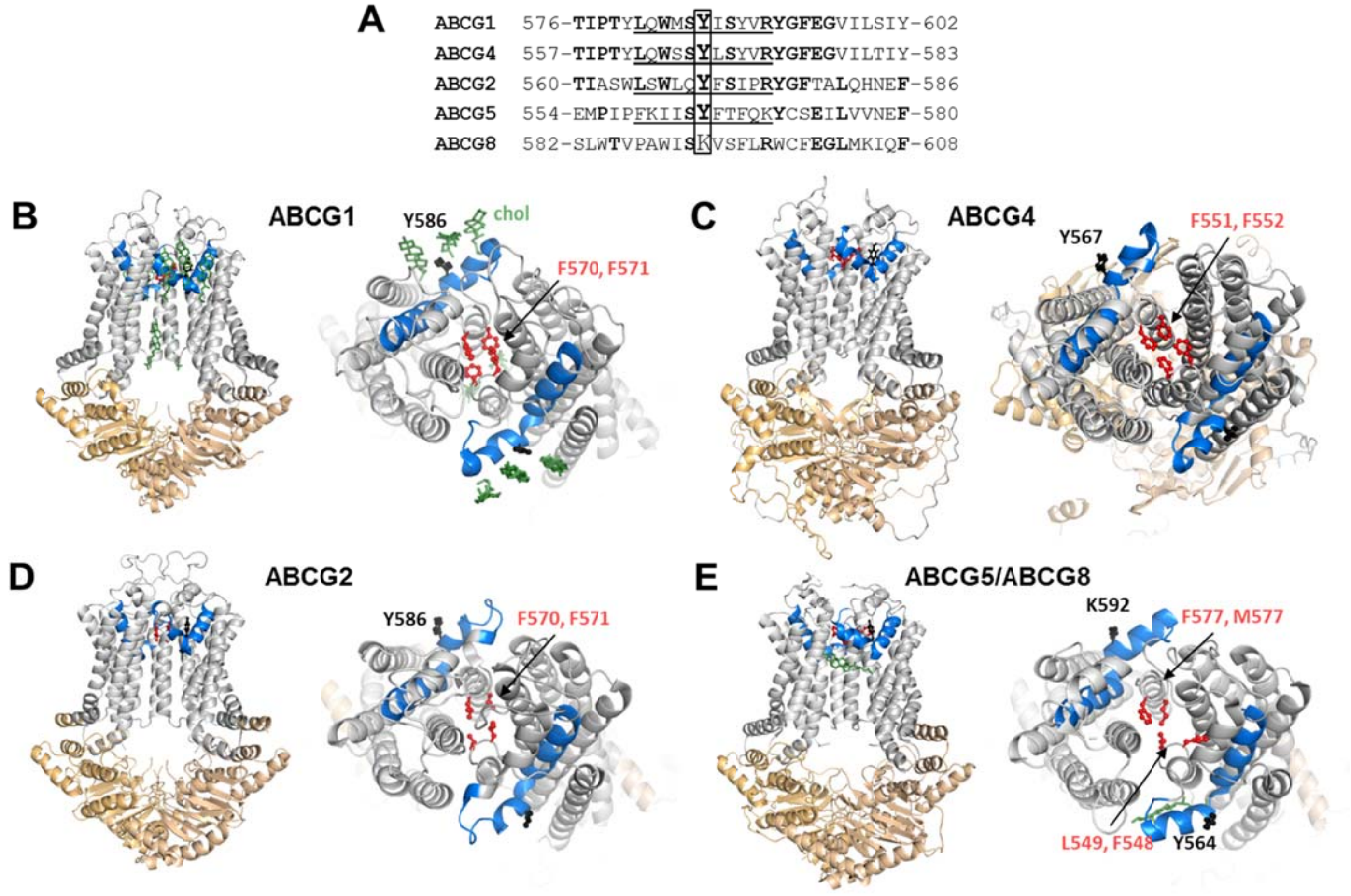

Supplementary Figure S4. Structual comparison of ABCG 1, ABCG4, ABCG2, and ABCG5/ABCG8. (A) Sequence alignment of the reentry regions based on structural alignments. CRAC motifs are underscored. Residues identical in two or three sequences are in bold. (B-E) Structures of ABCG1 (PDBID: 7r8d) [7], ABCG4 (https://3dbeacon.hegelab.org/struct/hege-abc-0002) [9], ABCG2 (PDBID: 6hco) [3], and ABCG5/ABCG8 (PDBID: 6cub) [2] are shown. NBD and TMD are colored with orange and grey colors, respectively. The reentry G-loop is indicated in blue. Tyrosine residues corresponding to ABCG1 Y586 are shown with black sticks and balls, as well as K592 in ABCG8, which lacks a CRAC motif in the G-loop. Residues corresponding to ABCG2 Leu-valve are represented by red sticks and balls. Green sticks indicate cholesterol molecules.
